# Supplementary material for: Pyruvate Dehydrogenase Kinase 1 inhibition mediated oxidative phosphorylation enhancement in cartilage promotes osteoarthritis progression
Source: BMC Musculoskelet Disord. 2023 Jul 20;24:597. doi: 10.1186/s12891-023-06585-6 (PMC10357736; doi:10.1186/s12891-023-06585-6)
Supplement: Supplementary file 1 — Additional file 1. [file 12891_2023_6585_MOESM1_ESM.zip › BP.Gsea.1653623667859/GOBP_HEAD_MORPHOGENESIS.html]

Details for gene set GOBP\_HEAD\_MORPHOGENESIS[GSEA]

|  || Dataset | 55457\_collapsed\_to\_symbols.cls.cls #OA\_versus\_normal.cls.cls #OA\_versus\_normal\_repos |
| Phenotype | cls.cls#OA\_versus\_normal\_repos |
| Upregulated in class | normal |
| GeneSet | GOBP\_HEAD\_MORPHOGENESIS |
| Enrichment Score (ES) | -0.51772416 |
| Normalized Enrichment Score (NES) | -1.7341726 |
| Nominal p-value | 0.016207455 |
| FDR q-value | 0.12131042 |
| FWER p-Value | 1.0 |
Table: GSEA Results Summary

  

Fig 1: Enrichment plot: GOBP\_HEAD\_MORPHOGENESIS      
 Profile of the Running ES Score & Positions of GeneSet Members on the Rank Ordered List

  

| SYMBOL | TITLE | RANK IN GENE LIST | RANK METRIC SCORE | RUNNING ES | CORE ENRICHMENT || 1 | RRAS | RAS related [Source:HGNC Symbol;Acc:HGNC:10447] | 505 | 0.658 | 0.0252 | No |
| 2 | RAB3GAP1 | RAB3 GTPase activating protein catalytic subunit 1 [Source:HGNC Symbol;Acc:HGNC:17063] | 1766 | 0.389 | -0.0347 | No |
| 3 | WNT3 | Wnt family member 3 [Source:HGNC Symbol;Acc:HGNC:12782] | 2003 | 0.360 | -0.0177 | No |
| 4 | PDGFRA | platelet derived growth factor receptor alpha [Source:HGNC Symbol;Acc:HGNC:8803] | 2943 | 0.256 | -0.0656 | No |
| 5 | SGPL1 | sphingosine-1-phosphate lyase 1 [Source:HGNC Symbol;Acc:HGNC:10817] | 3462 | 0.206 | -0.0857 | No |
| 6 | TBX1 | T-box transcription factor 1 [Source:HGNC Symbol;Acc:HGNC:11592] | 4679 | 0.098 | -0.1706 | No |
| 7 | DLX5 | distal-less homeobox 5 [Source:HGNC Symbol;Acc:HGNC:2918] | 4700 | 0.096 | -0.1628 | No |
| 8 | ATP6AP2 | ATPase H+ transporting accessory protein 2 [Source:HGNC Symbol;Acc:HGNC:18305] | 5203 | 0.056 | -0.1963 | No |
| 9 | TGFB3 | transforming growth factor beta 3 [Source:HGNC Symbol;Acc:HGNC:11769] | 6293 | -0.027 | -0.2782 | No |
| 10 | DKK1 | dickkopf WNT signaling pathway inhibitor 1 [Source:HGNC Symbol;Acc:HGNC:2891] | 6392 | -0.034 | -0.2825 | No |
| 11 | MMP2 | matrix metallopeptidase 2 [Source:HGNC Symbol;Acc:HGNC:7166] | 7037 | -0.082 | -0.3245 | No |
| 12 | STRA6 | signaling receptor and transporter of retinol STRA6 [Source:HGNC Symbol;Acc:HGNC:30650] | 7107 | -0.089 | -0.3212 | No |
| 13 | SKI | SKI proto-oncogene [Source:HGNC Symbol;Acc:HGNC:10896] | 7349 | -0.106 | -0.3295 | No |
| 14 | LEF1 | lymphoid enhancer binding factor 1 [Source:HGNC Symbol;Acc:HGNC:6551] | 7467 | -0.116 | -0.3273 | No |
| 15 | ASPH | aspartate beta-hydroxylase [Source:HGNC Symbol;Acc:HGNC:757] | 7533 | -0.121 | -0.3205 | No |
| 16 | COL1A1 | collagen type I alpha 1 chain [Source:HGNC Symbol;Acc:HGNC:2197] | 8133 | -0.165 | -0.3509 | No |
| 17 | MYH3 | myosin heavy chain 3 [Source:HGNC Symbol;Acc:HGNC:7573] | 8258 | -0.176 | -0.3433 | No |
| 18 | CLDN5 | claudin 5 [Source:HGNC Symbol;Acc:HGNC:2047] | 9816 | -0.302 | -0.4347 | No |
| 19 | IHH | Indian hedgehog signaling molecule [Source:HGNC Symbol;Acc:HGNC:5956] | 10886 | -0.415 | -0.4771 | Yes |
| 20 | PLEKHA1 | pleckstrin homology domain containing A1 [Source:HGNC Symbol;Acc:HGNC:14335] | 10899 | -0.416 | -0.4373 | Yes |
| 21 | PTPN11 | protein tyrosine phosphatase non-receptor type 11 [Source:HGNC Symbol;Acc:HGNC:9644] | 11260 | -0.465 | -0.4197 | Yes |
| 22 | EP300 | E1A binding protein p300 [Source:HGNC Symbol;Acc:HGNC:3373] | 11741 | -0.540 | -0.4042 | Yes |
| 23 | PAX9 | paired box 9 [Source:HGNC Symbol;Acc:HGNC:8623] | 12028 | -0.600 | -0.3677 | Yes |
| 24 | MSX1 | msh homeobox 1 [Source:HGNC Symbol;Acc:HGNC:7391] | 12054 | -0.609 | -0.3101 | Yes |
| 25 | ARID5B | AT-rich interaction domain 5B [Source:HGNC Symbol;Acc:HGNC:17362] | 12187 | -0.640 | -0.2577 | Yes |
| 26 | CRISPLD2 | cysteine rich secretory protein LCCL domain containing 2 [Source:HGNC Symbol;Acc:HGNC:25248] | 12188 | -0.641 | -0.1949 | Yes |
| 27 | NIPBL | NIPBL cohesin loading factor [Source:HGNC Symbol;Acc:HGNC:28862] | 12385 | -0.703 | -0.1413 | Yes |
| 28 | ANKRD11 | ankyrin repeat domain 11 [Source:HGNC Symbol;Acc:HGNC:21316] | 12738 | -0.902 | -0.0804 | Yes |
| 29 | TIPARP | TCDD inducible poly(ADP-ribose) polymerase [Source:HGNC Symbol;Acc:HGNC:23696] | 12789 | -0.950 | 0.0086 | Yes |
Table: GSEA details [plain text format]

  

Fig 2: GOBP\_HEAD\_MORPHOGENESIS      
 Blue-Pink O' Gram in the Space of the Analyzed GeneSet

  

Fig 3: GOBP\_HEAD\_MORPHOGENESIS: Random ES distribution      
 Gene set null distribution of ES for **GOBP\_HEAD\_MORPHOGENESIS**
